# Progress in etiological diagnosis of viral meningitis

**DOI:** 10.3389/fneur.2023.1193834

**Published:** 2023-07-31

**Authors:** Hongyan Xu, Peng Chen, Shihan Guo, Xiaokai Shen, Yun Lu

**Affiliations:** ^1^Emergency Department, Hospital of Chengdu University of Traditional Chinese Medicine, Chengdu, China; ^2^Department of General Practice, The 900th Hospital of Joint Logistic Support Force, PLA, Fuzong Clinical College of Fujian Medical University, Fuzhou, Fujian, China; ^3^Department of Dermatology, Xiangya Hospital, Central South University, Changsha, China; ^4^Department of Neurology, Xiangya Hospital, Central South University, Changsha, China

**Keywords:** viral meningitis, etiology, diagnosis, PCR, NGS

## Abstract

In recent years, with the rapid development of molecular biology techniques such as polymerase chain reaction and molecular biochip, the etiological diagnosis of viral encephalitis has a very big step forward. At present, the etiological examination of viral meningitis mainly includes virus isolation, serological detection and molecular biological nucleic acid detection. This article reviews the progress in etiological diagnosis of viral meningitis.

## 1. Introduction

Viral meningitis is the most common infection of the central nervous system and is caused by viral invasion of the meninges (1, 2). It is common in immunocompromised people such as children and the elderly (3, 4). In addition, host genetic inheritance would also influence viral invasion (5–7). For instance, it directly influences the host immune response to viral infection (such as innate immune response and adaptive immune response), or indirectly influences susceptibility to viral infection by affecting other factors such as age, sex and comorbidities, resulting in differences in disease severity and outcome. Currently, the causative agent of viral meningitis is mainly human enterovirus (EV) (8–11). The virus has more than 60 different subtypes, including poliovirus, coxsackievirus A, coxsackievirus B, and echovirus (12–14). And others such as herpes simplex virus (HSV) and varicella-zoster virus (VZV) are also common pathogens causing this disease (15–17), but mumps virus and influenza virus are relatively rare. During the COVID pandemic, despite being reported, the incident rate of meningitis caused by SARS-CoV-2 seems extremely low.

Laboratory diagnostic methods include microscopic examination of clinical specimens, viral culture, serological studies, immunodetection of viral or viral antigens and even sequence reading of DNA or RNA (18). Early sample sizes were limited and virus concentrations were low, severely hindering accurate detection of pathogens by microscopy. The viruses invaded the central nervous system mainly by three routes, the blood–brain barrier penetration, axonal transport and Trojan-Horse-Mediated entry, leaving different traces detectable by different methods (19). At the same time, high mutation rates of EVs (20) and long viral shedding times necessitate continued surveillance to identify changes in EV infection in the central nervous system and transmission of EVs (21). Moreover, EV-71 is difficult to detect from CSF and can usually be detected by polymerase chain reaction (PCR) from stool and throat swabs (22–25). Therefore, choosing an optimally rapid and accurate method is of great importance. After a systematic search of the literature, we found that there is currently no article that comprehensively and in detail discusses the etiological diagnostic techniques for viral meningitis. Therefore, it is our aim to comprehensively analyze the existing etiological diagnostic techniques for viral meningitis, hoping to provide some reference when clinicians track the cause of meningitis.

## 2. Methods

This study was conducted using a comprehensive search of PubMed and Web of Science databases. The keywords used to search for information were meningitis, virus, etiology, and diagnosis. The articles studied are currently published and the criteria included in this paper are: (1) the disease studied is viral meningitis; (2) the subjects are human; and (3) diagnostic techniques of etiology. Meanwhile, exclusion criteria were: (1) clinical symptoms of viral meningitis and routine, biochemical, and cytological examination of cerebrospinal fluid; and (2) research on animal experiments. Data collected, registered, and reported for this study were independent of seasonal and geographical factors, and the study considered publications relevant to patients of all age groups. The included articles were perused and summarized, and the retrieved content was used for descriptive analysis. The relevant information on disease etiology, etiology and its diagnostic methods were extracted and documented from the selected publications. And a narrative presentation followed the compilation of these data.

## 3. Etiological examination

### 3.1. Microscopic examination

Transmission electron microscopy (EM) is a method that determines the pathogens by examining the cellular morphology of specimens. It lacks specificity for any specific group of viruses, which broadens the clinical outcomes and delays treatment (26, 27). The main limitation of this method is its relatively high detection limit (10^6^–10^7^ particles/mL) (28), requiring virus material concentration. However, the concentration process can lead to viral loss. Although the application of electron microscopy in the examination of coronaviruses and enteric viruses has been advancing in recent years (29), it relies on limited cerebrospinal fluid (CSF) samples, necessitating the search for an effective universal method for virus concentration.

### 3.2. Virus isolation

Judging accurate pathogens by performing virus isolation from clinical specimens is the “gold standard” for diagnosis since the 1900 s, and this phenomenon has been going on for decades (30). For example, EV and HSV are isolated from cerebrospinal fluid, while poliovirus and rotavirus are often isolated from feces (31). Hank’s solution can be used to extract the virus from clinical samples, and then proliferate the virus through virus culture and carry out subsequent identification or detection and other operations. Virus isolation by cell culture followed by the cytopathic effect (CPE) assay is the most commonly used viral identification method in biological samples (32). For example, the classical approach to diagnose EV infection is to isolate the virus from clinical specimens by cell culture followed by neutralization assays to determine serotypes (31). A prospective study by Petrousová et al. (33) included 34 patients with aseptic neurological infections. EM detected CSF from all patients and virus isolation was performed on all CSF samples, and the results showed that virus isolation was successful in 10 patients. It should be noted that virus isolation is now less used in the clinic, due to its low sensitivity and time-consuming nature (34–36).

### 3.3. Serologic testing

#### 3.3.1. Enzyme-linked immunosorbent assay

Enzyme-linked immunosorbent assay (ELISA) is a qualitative and quantitative detection method, using antigen–antibody binding specificity (37). One study measured CSF concentrations of tryptophan (Trp) and kynurenine (Kyn) using ELISA in 76 patients and found that Kyn concentrations and Kyn/Trp ratio were highly increased in viral CNS infections, while patients with autoimmune neuroinflammatory and non-inflammatory diseases showed low concentrations (38). Day et al. (39) performed ELISA to detect enterovirus-specific IgM in serum samples from 557 patients clinically diagnosed with meningitis or encephalitis and found that the detection rate was much higher than the virus isolation rate. Detection of EV IgM by ELISA is therefore a more sensitive, economical, and rapid diagnostic method than virus isolation and is not affected by viral viability (40, 41). For some viruses, including Epstein–Barr virus (EBV), potentially associated with neurological diseases such as multiple sclerosis (MS), activation of a polyclonal response at the intrathecal level has been described (42).

Beforehand, the role of EBV-specific humoral immune response in pathogenetic process of MS remained unknown, since the attempt to detect EBV-infected B cells in MS brain lesions had failed (43–45). In contrast to previous publications which measured anti-EBV antibodies by chemiluminescent immunoassays, Castellazzi et al. (42) assessed intrathecal and serum levels of anti-EBV IgG by ELISA methods for the first time in CSF and serum samples of both MS patients and controls. The study provided an avenue for ELISA to investigate these potentially viral-related neurological diseases.

#### 3.3.2. Immunofluorescence

Immunofluorescence is a technique for the detection of microbial samples by using fluorescence microscopy (46). Pierro et al. (47) used indirect immunofluorescence to detect specific antibodies during the assessment of the kinetics of anti-TOSV antibodies over time in 41 patients diagnosed with TOSV meningitis or meningoencephalitis in northeast Italy. Meqdam et al. (48) detected the presence of EVs using viral culture and indirect immunofluorescence in the study of the prevalence of enteroviral meningitis and its relationship with clinical outcomes in northern Jordan. It has been shown that measurement of calcitonin using antibody-conjugated fluorospheres can differentiate atypical bacterial meningitis from viral encephalitis in children (49). Immunofluorescence microscopy enables single-cell analysis of samples and preserves spatial information within cells as well as throughout the culture, and can be paired with image analysis tools to localize to the virus or detect expression of associated viruses at the single-cell level, but this technique is only useful in the absence of infected cells (50).

#### 3.3.3. Hemagglutination inhibition test

Hemagglutination inhibition test is to use the nature that the virus concentration is positively correlated with the degree of hemagglutination of red blood cells, add specific antibodies to the suspension of the virus, then prevent the contact between red blood cell surface receptors and virus particles or their hemagglutinins, and thus inhibit the hemagglutination of red blood cells. Indirect hemagglutination inhibition testing was found to be very sensitive for antibody detection in convalescent animals as well as in chronic lymphocytic choriomeningitis virus-infected mice in a study of mice infected with chronic lymphocytic choriomeningitis virus isolated in 1976 (51). During the 1999 Russian outbreak of serous meningitis and meningoencephalitis, two West Nile viruses, Ast 986 and LEIV 27889 Vig, were confirmed to be the main causes using convalescent sera tested in a hemagglutination inhibition test (52). However, because the hemagglutination inhibition test has a strict choice for red blood cell types, temperature during reaction and PH when detecting the virus of viral meningitis, it is gradually replaced by ELISA, so hemagglutination inhibition test is rarely used to diagnose viral meningitis at present.

### 3.4. Molecular biology nucleic acid test

#### 3.4.1. Polymerase chain reaction

Molecular biological nucleic acid testing for viral meningitis is performed primarily by PCR. PCR is a widely used method for multiplex replication of specific DNA fragments in molecular biology (53). Using PCR, a single replication (or more) of a DNA sequence is exponentially amplified, resulting in the replication of thousands of specific DNA fragments. Simultaneously, PCR is a common and often indispensable technique widely used in medical laboratory and clinical laboratory research, including biomedical research and criminal forensics (54). In the acute phase of infection, nucleic acid amplification is the preferred method for the diagnosis of viral meningitis in CSF samples (55, 56). Because molecular methods are rapid and sensitive, unlike traditional methods, such as virus isolation by cell culture, they are not affected by the viability of the virus in clinical specimens. Development of PCR technology has improved specificity and time taken to perform testing, including real-time PCR, reverse transcription PCR, and multiplex nested PCR (57–60).

##### 3.4.1.1. Real-time polymerase chain reaction

Real-time polymerase chain reaction, also known as real-time fluorescence quantitative polymerase chain reaction (qPCR), is a molecular biology laboratory technique based on PCR. It uses fluorescence signal accumulation to monitor the amplification of target DNA molecules during PCR in real time, rather than at their ends as in traditional PCR. Khumalo et al. (61) developed two qPCRs for the detection of six common pathogens of community-acquired bacterial and viral meningitis in South African children and showed that none of the cases that tested positive by viral qPCR were confirmed to be caused by bacteria in cerebrospinal fluid cultures. It can be seen that in this population, qPCR use against common pathogens has achieved good results. One study performed conventional or multiplex real-time PCR on 373 CSF samples from patients with clinically suspected neuroviral infections and found an increased frequency of CSF positive samples for human adenovirus (HAdV) after changing from conventional PCR to multiplex qPCR (62). Recently, Huang et al. developed a new fluorescent PCR technology called “MeltArray” to fill the technical gap that has long existed between low-order PCR and high-throughput detection, which can detect dozens of targets per reaction in a qPCR thermocycler (63), and also take only a few hours to produce results (22). This new assay has now been used in clinical practice because of its combined advantages of diversity, versatility, simplicity, and accessibility.

##### 3.4.1.2. Reverse transcription polymerase chain reaction

Reverse transcription polymerase chain reaction (RT-PCR) is a variant of PCR and is a common technique for detecting RNA expression in molecular biology. RT-PCR is used to qualitatively detect gene expression by creating complementary DNA (cDNA) from RNA. Traditional PCR techniques allow exponential amplification of the target DNA sequence. RT-PCR is the reverse transcription of RNA of interest into its complementary DNA using reverse transcriptase and is mostly used to clone expressed genes. Subsequently, newly synthesized cDNA was amplified with traditional PCR. In addition to qualitative studies of gene expression, RT-PCR can be used for quantitative detection of RNA. Raouf et al. (2) collected patients with suspected meningitis from Alshatby University and Alexandria Fever Hospitals over a specific time period, from whom 94 were randomly selected for RT-PCR, and 7/94 (7.45%) non-polio enteroviruses (NPEVs) were detected. One case describes a neonate who presented with two episodes of viral meningitis within 1 month, both of which were nonspecific in clinical features, and was tested by RT-PCR for EV and human paracovirus (HPeV), respectively (64). For the detection of Toscana Virus, the detection rate was significantly improved in 2007 by transitioning from viral culture to real-time RT-PCR (56). Reverse transcription quantitative PCR (RT-qPCR) has been widely used in molecular biology and virology due to its advantages of rapidity, sensitivity and reproducibility (65).

##### 3.4.1.3. Nested polymerase chain reaction

Nested polymerase chain reaction (nPCR) is a modification of polymerase chain reaction that can reduce non-specific binding in products. Nested PCR involves two sets of primers for two consecutive polymerase chain reactions, and the second set of primers can only amplify the expected product from the first round, but not non-specific products. It therefore allows for a greater number of cycles while reducing non-specific products. Drago et al. (66) compared nPCR and qPCR techniques for the detection of Cytomegalovirus, HSV-1, and Epstein–Barr virus (EBV) in CSF of HIV patients. Then it can be found that nPCR is as sensitive but more time-consuming as qPCR in diagnosing herpes virus infections of the CNS. Moreover, FilmArray meningitis/encephalitis (ME) technology with multiplex molecular panel approved by FDA in 2015 is also based on nPCR principle, which has the efficacy of rapid (about 60 min) and comprehensive detection of selected meningitis and encephalitis pathogens and is currently used as an auxiliary means for the diagnosis of CNS infections (67–69).

The combined technique of qPCR and RT-PCR is called quantitative RT-PCR, usually referred to as qRT-PCR, which is considered to be the most powerful and sensitive method for detecting RNA levels relative to other RNA quantification methods, such as northern blot. It is commonly used for expression analysis of single or multiple genes, as well as for identifying expression patterns in infections and diseases. But the qRT-PCR assay was not sensitive enough to detect samples with low viral loads, especially in CSF of patients. Shen et al. (70) proposed a highly sensitive real-time nested RT-PCR (RTN RT-PCR) assay for the detection of human EVs. The clinical diagnostic efficacy of RTN RT-PCR and qRT-PCR was tested and compared by 140 cerebrospinal fluid and stool samples. RTN RT-PCR was found to be more sensitive than qRT-PCR for detection of human enteroviruses. And it is consistent with Farshadpour and Taherkhani (11).

Because PCR not only shortens the time and reduces the risk of contamination in the detection of pathogens (55), but also has a high sensitivity, it is now widely used in clinical practice. In particular, multiplex PCR, also known as multiplex primer PCR, has become a commonly used nucleic acid detection technique (69, 71–73). However, the rapid routine molecular diagnosis achieved by PCR is limited to the detection of known infectious agents (74, 75), so it needs to be diagnosed with more other detection methods when necessary.

#### 3.4.2. NGS technology

NGS technology is also called massive parallel sequencing (MPS) or high-throughput sequencing (HTS), and there are two main methods for the detection of pathogenic microorganisms, including metagenomic metagenomic Next Generation Sequencing (mNGS) and targeted sequencing (tNGS).

##### 3.4.2.1. Metagenomic Next Generation Sequencing

Metagenomic Next Generation Sequencing (mNGS) is a novel and promising method that can simultaneously and unbiased identify all microorganisms in human samples (75–77). Yu et al. (78) found that mNGS achieved good efficacy in diagnosing free DNA on viral CNS infection. Piantadosi et al. (79) performed comprehensive mNGS on 68 prospectively enrolled patients with known (*n* = 44) or suspected (*n* = 24) CNS viral infections from a single New England center and assessed enhanced methods to improve CNS pathogen detection and identify pathogens traditionally not identified by nucleic acid testing. Leon et al. (75) applied mNGS and CSF pan-viral serology (VirScan) to detect EVs in CSF and found that VirScan’s mNGS significantly improved CNS detection of EVs compared with qRT-PCR. While Anh et al. detected viruses in 204 CSF samples from patients with acute central nervous system infections registered from hospitals in Vietnam from 2012 to 2016 using mNGS, eight viruses were detected in 107/204 (52.4%) CSF samples. After confirmation by virus-specific PCR, the detection rate decreased to 30/204 (14.7%). These results indicate that mNGS will overdetect pathogens, which are considered to be caused by unavoidable reagent contamination at present (80). However, reducing cellular DNA concentrations in CSF may reduce the sensitivity of mNGS for the detection of DNA viruses (e.g., HSV) (81, 82). At the same time, Xing et al. (83) found that mNGS could not significantly predict meningitis caused by RNA viruses such as EV and Japanese encephalitis virus, therefore, it is necessary to improve the DNA/RNA co-extraction method and sequence DNA and RNA to improve the virus detection rate. And host information in mNGS detection accounts for more than 90% of the sequencing data volume, while the signal of pathogenic microorganisms is relatively weak, so the requirements for detection sensitivity are high. Because of its high output and resolution, mNGS not only provides us with rich genetic information, but also greatly shortens the cost and time of sequencing.

##### 3.4.2.2. Targeted sequencing

Targeted sequencing (tNGS) refers to the sequencing of specific regions using PCR or probe hybridization methods. Korimbocus et al. (34) successfully identified herpes virus (HSV-1, HSV-2) and cytomegalovirus; all serotypes of human EV; and five flaviviruses (West Nile Virus, Dengue Virus, and Langart Virus) using probe hybridization. If only pathogenic microorganism nucleic acids are sequenced during sequencing, the detection sensitivity of pathogenic microorganisms can be greatly improved while reducing the sequencing cost. Furthermore, resistance genes were also detected while pathogen detection was done.

Major pathogens can be detected in most samples by metagenomics, but the results obtained are affected by low concentrations of pathogens, massive contamination and collation, and the range of reference databases for bioinformatics analysis (74, 84), so techniques such as PCR maybe need to be used to assist in diagnosis.

## 4. Discussion

Nowadays, viruses are increasingly emerging as important etiologies of meningitis (85–87). Although the type of disease can be initially identified based on the patient’s physical signs and routine biochemical tests, accurate determination of the pathogen cannot solely rely on gram stain and biochemical parameters. Therefore, PCR or NGS techniques are generally employed for pathogenic diagnosis, which is consistent with previous literature (8, 9, 88).

Now, molecular techniques are the gold standard for detecting the etiology of viral meningitis, not only improving the detection rate of pathogens without affecting viral viability in specimens (55), but also reducing the use of unnecessary antibiotics and length of hospital stay (2). Previously, virus isolation (tissue culture) from CSF, blood, or urine was the gold standard for the diagnosis of many viral pathogens causing meningitis, however, this process was slow, expensive, and not always sensitive. Therefore, many laboratories now offer cerebrospinal fluid PCR services that include EV and HSV, and can also choose to detect cytomegalovirus and VZV. RT-PCR detection of enteroviruses is more sensitive (faster) than CSF culture, and PCR detection of herpesvirus is equally effective in improving the accuracy and speed of diagnosis. The development of PCR technology has improved specificity and reduced the time required to perform the test, and PCR, as a rapid, sensitive, and specific diagnostic measure, will play a more important role in the diagnosis of meningitis (80). Serologic testing for multiple other pathogens is required based on clinical features and exposure history when viral PCR is negative for aseptic meningitis. Then mNGS can be a meaningful method (16). So CSF mNGS combined with routine tests (including serological tests and sample types other than cerebrospinal fluid) has the highest diagnostic yield (72, 77, 89).

Reliable and appropriate diagnostic techniques are urgently needed for patients suspected of having viral meningitis, particularly in coma, while communicating effectively with the patient’s family. If direct selection of PCR or NGS techniques is unreasonable, it is like serum and cerebrospinal fluid immunoglobulin M antibody detection is the preferred diagnostic test for arboviruses (90), which requires clinicians to have a certain level of clinician knowledge in selecting diagnostic techniques. Our article just provides a theoretical foundation for this. Previous articles only briefly touched upon diagnostic techniques, lacking in-depth descriptions and analyses of pathogenic diagnostic tools. Our article offers a more comprehensive depiction of the available pathogenic diagnostic techniques and advancements in viral meningitis, provides guidance for the selection of etiological diagnostic techniques, and brings theoretical support to clinicians for the selection of test methods for patients.

However, our paper solely starts with etiological diagnosis methods, and does not analyze the signs and routine biochemical tests of patients, so needs to be used in combination with other literatures, guidelines and clinical experience. At the same time, considering the complex pathophysiology of SARS-CoV-2, it remains difficult for choosing the optimal methods (91). Finally, the economic level of patients is not considered in the technical selection, NGS technology costs are relatively high, and is generally not routinely used in clinical practice. Perhaps the subsequent clinical selection can be further compared according to the economic level.

## 5. Conclusion

Viral meningitis remains a major human medical problem, and diagnostic techniques for etiology are also continuously innovating. Choosing rational diagnostic techniques to understand the etiology and pathogenesis of this disease allows for faster therapeutic intervention, which will help to improve outcomes. In this COVID pandemic, though neuroinvasion has been rarely reported, it is worth noted that this rare neuroinvasion might be explained by the inappropriate detection methods. In the future, etiology diagnosis could be made in a more rapid and precise way, as the progress of etiology diagnose technique, together with the advance of the understanding on the pathology of infective agents.

## Author contributions

HX, PC, and SG worked together to collect the data and write the manuscript, with subsequent revisions and improvements. XS revised and polished the manuscript. YL was responsible for the conception of the work, as well as the drafting of the work and constructive comments on the content of the article, paying close attention to all aspects of the article to ensure the accuracy and completeness of any part of the article. All authors contributed to the manuscript and approved the version as submitted.

## Conflict of interest

The authors declare that the research was conducted in the absence of any commercial or financial relationships that could be construed as a potential conflict of interest.

## Publisher’s note

All claims expressed in this article are solely those of the authors and do not necessarily represent those of their affiliated organizations, or those of the publisher, the editors and the reviewers. Any product that may be evaluated in this article, or claim that may be made by its manufacturer, is not guaranteed or endorsed by the publisher.
